# Small Molecule NF-κB Pathway Inhibitors in Clinic

**DOI:** 10.3390/ijms21145164

**Published:** 2020-07-21

**Authors:** Venkataramanan Ramadass, Thamilselvan Vaiyapuri, Vinay Tergaonkar

**Affiliations:** 1 Institute of Molecular and Cell Biology (IMCB), Singapore 138673, Singapore; vinayt@imcb.a-star.edu.sg; 2 Department of Pathology, NUS, Singapore 117597, Singapore

**Keywords:** NF-κB pathway, small molecules, clinical trials, anti-cancer, anti-inflammatory, autoimmune disease

## Abstract

Nuclear factor kappa B (NF-κB) signaling is implicated in all major human chronic diseases, with its role in transcription of hundreds of gene well established in the literature. This has propelled research into targeting the NF-κB pathways for modulating expression of those genes and the diseases mediated by them. In-spite of the critical, but often promiscuous role played by this pathway and the inhibition causing adverse drug reaction, currently many biologics, macromolecules, and small molecules that modulate this pathway are in the market or in clinical trials. Furthermore, many marketed drugs that were later found to also have NF-κB targeting activity were repurposed for new therapeutic interventions. Despite the rising importance of biologics in drug discovery, small molecules got around 76% of US-FDA (Food and Drug Administration-US) approval in the last decade. This encouraged us to review information regarding clinically relevant small molecule inhibitors of the NF-κB pathway from cell surface receptor stimulation to nuclear signaling. We have also highlighted the underexplored targets in this pathway that have potential to succeed in clinic.

## 1. Introduction

Nuclear factor kappa B (NF-κB) signaling has been well studied for more than three decades from the first report by Sen et al. [1] in 1986. It is implicated in both normal physiology [2,3] and in the development of a multitude of diseases [4] ranging from inflammation [5,6], cancer [7,8,9] to autoimmune disorders [10,11]. Thus, it is not surprising that many researcher groups are actively pursuing efforts to modulate this pathway for therapeutic intervention. Despite the vast knowledge for this pathway and several compounds in preclinical stage still there is a dearth of clinically proven molecules. As a case in point, Gilmore et.al. [12] enlisted 785 inhibitors of NF-κB signaling in 2006 but only few of them did even reach clinical trials. In this review, we aim to analyze the small molecule inhibitors of NF-κB pathways that were taken into clinical trials.

NF-κB family consists of five transcription factors: NF-κB1/p50, NF-κB2/p52, RelA/p65, RelB and c-Rel that homodimerize or heterodimerize to 15 NF-κB complexes [13]. These are mainly found in the cytoplasm of resting cells and upon activation they translocate to the nucleus for transcription which in-turn leads to direct or indirect activation or repression of hundreds of genes. Notwithstanding their ubiquitous nature, they show high contextual diversity for transcription based on the tissue type and specific biological circumstances [14].

**Figure 1:**
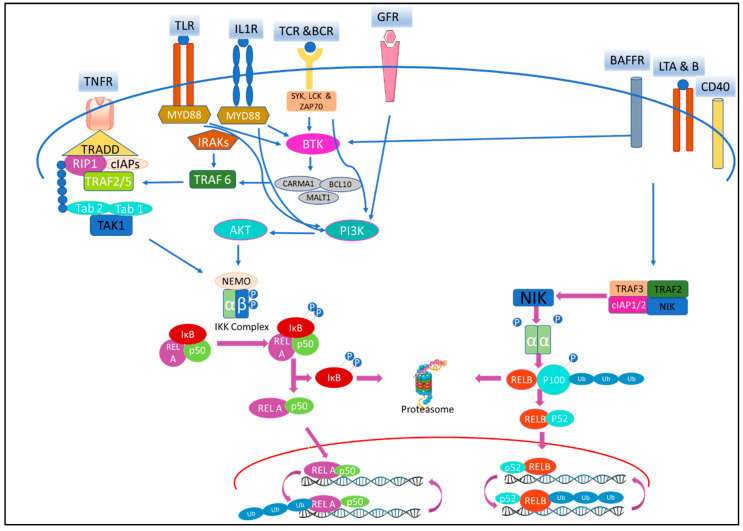
Schematic diagram of the canonical and non-canonical Nuclear factor kappa B (NF-κB) signaling pathways. Canonical NF-κB signaling cascade is initiated at the cell membrane through tumor necrosis factor –α receptor (TNFR), interleukin1 receptor (IL1R), toll-like receptor (TLR), T-cell receptors (TCR), B-cell receptors (BCR), and growth factor receptors (GFR) while the non-canonical pathway can be stimulated via receptors such as lymphotoxin α and β (LTA and B), cluster of differentiation 40 (CD40) and B-cell activating factor (BAFF). Upon stimulation of the receptor, the canonical pathway is mediated through adapter proteins and kinases that eventually activate the IκB kinase (IKK) complex via activation of transforming growth factor β-activated kinase 1 (TAK1). Similarly, PI3K mediates signaling from GFR, TLRs, TCR, BCR, and cytokines feeds into activation of IKK via phosphorylation of AKT (protein kinase B) and IKKα. Subsequently, polyubiquitination and site-specific phosphorylation of IκB protein via IKK complex leads to its proteasomal degradation. The released NF-κB dimers translocate to the nucleus. Meanwhile, during the activation of the non-canonical pathway, TNF receptor-associated factor (TRAF) and cellular inhibitor of apoptosis (c-IAP) are recruited to the receptor as TRAF2/TRAF3/c-IAP1/2 complex. TRAF2/TRAF3 undergo proteasomal degradation allowing NF-κB-inducing kinase (NIK) levels to be stabilized. NIK then phosphorylates IKKα homodimer. Activated IKKα homodimer phosphorylates p100, leading to the partial degradation of p100 to p52. RelB/p52 heterodimers translocate to nucleus for further transcription.

The NF-κB pathway can be triggered by diverse stimuli that includes inflammatory cytokines, immune signals and antigens, microbial products and stress signals [15]. Upon stimulation, either the canonical or the non-canonical pathway (Figure 1) is activated, depending on the type of Inhibitor of κB (IκB) protein or IκB kinase (IKK) complex involved. The biology of this signaling cascade is well reviewed in the literature [16,17]. On stimulation of the receptor, canonical pathway mediated through adapter proteins and kinases, activates IKK complex that comprises of IKKα/β/γ. Polyubiquitination and site-specific phosphorylation subsequently lead to proteasomal degradation of IκB protein and release of NF-κB dimers for translocation to the nucleus. Furthermore, the pathway mediated by NF-κB1/p50, also involves partial degradation of p105 to p50. Non-canonical pathway [18,19], on stimulation recruit tumor necrosis factor (TNF) receptor-associated factor (TRAF) and cellular inhibitor of apoptosis (c-IAP) as TRAF2/TRAF3/c-IAP1/2 complex. NF-κB-inducing kinase (NIK), that is constitutively bound to the complex, on activation dissociates, gets stabilized and phosphorylate IKKα homodimer. This leads to partial degradation of p100 to p52 and the RelB/p52 heterodimer translocate to nucleus for further transcription [20].

At homeostasis, NF-κB pathways are essential for regulating host defense responses against stress, injury and infection [13]. The pathways that are normally transient and self-limiting, have been shown to be excessively or constitutively active under disease condition. Dysregulation of NF-κB leads to the pathogeneses of cancer, inflammation, diabetes, and autoimmune disorders [13,17,18,21]. It is also implicated in the refraction of current therapies in both oncological [22,23] and non-oncological conditions [24]. These findings have generated very high interest in targeting the NF-κB pathway, but the real challenge exists in overcoming its ubiquitous physiological functions and have only contextual, tissue specific function [25,26,27].

**Figure 2:**
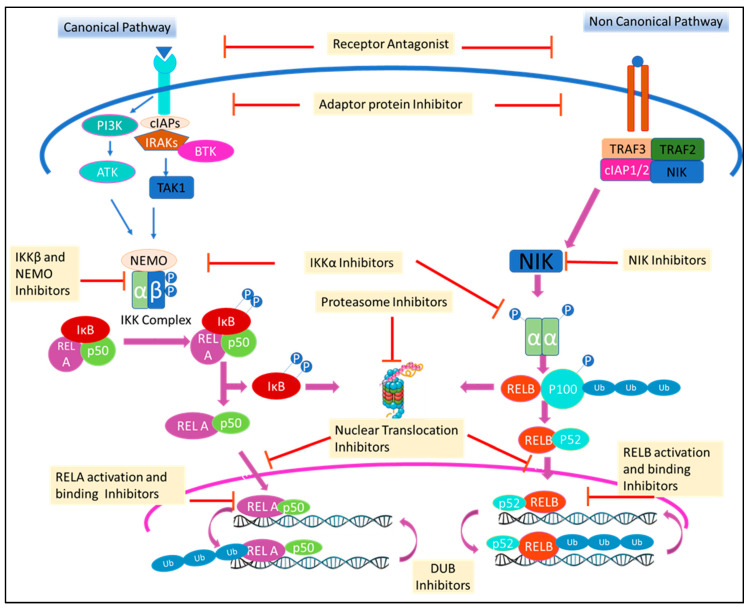
Canonical & non-canonical NF-κB pathway and the site of interventions currently being explored for therapeutic benefit.

NF-κB signaling cascades can be modulated at stages involving the receptor, IKK complex, gene transcription, post-translation or at any stage in between (Figure 2). This has been accomplished [4,28] using small molecules, peptides, oligonucleotides, monoclonal antibodies (mAbs), and small interfering RNA (siRNA). Among these drugs, small molecules are of special interest due to the ease of designing, synthesizing and amending in drug development research [29,30]. Though riddled with challenges of selectivity, multiple targets and adverse drug reaction (ADR) in clinic, modern school of thoughts now encourage a molecule to hit multiple targets for a desired efficacy and to avoid resistance [31]. Moreover, even with the rising importance of biologic drugs in drug discovery, 76% of US-FDA (Food and Drug Administration-US) approved drugs in the last decade were still small molecules [32]. This encouraged us to review information regarding clinically relevant small molecule inhibitors of the NF-κB pathway from cell membrane receptor stimulation to nuclear signaling. Apart from evaluating the successes and failures of these small molecule NF-κB inhibitors during clinical trials, we also reviewed drugs that have been re-purposed after being shown to have NF-κB activity. We have consciously excluded herbal extract that often involves multiple components, but included small molecules isolated from natural products. Furthermore, we have focused only on modalities that inhibit the pathway as it has seen more clinical success than those activating cascade. Special emphasis has been given to molecules that were withdrawn or discontinued and the probable reason, if the same is disclosed in public domain. Molecules in clinical trials held-up at a stage with no further reports are often taken as drop-outs, but we have cannot consider them to be withdrawn due to absence of any credible information.

## 2. Molecules that Inhibit Upstream IKK Complex in NF-κB Pathway

### 2.1. Inhibition of Cell Membrane Receptor Targets

NF-κB signaling cascade are mostly initiated at the cell membrane through tumor necrosis factor-α receptor (TNFR), interleukin1 receptor (IL1R), toll-like receptor (TLR), T-cell receptors (TCR), B-cell receptors (BCR), growth factor receptors and TNF receptor superfamily (TNFRSF), such as lymphotoxin (LT) α/β, cluster of differentiation (CD) 27, CD30, CD40, receptor activator of NF-κB (RANK), fibroblast growth factor-inducible 14 (Fn14), and B-cell activating factor (BAFF) receptors [33]. The cell surface receptors are most ideal targets to inhibit most of the pathways, but involved generally in protein–protein interaction (PPI), they lack binding sites for small molecules. They are mostly targeted by antibodies, siRNA, oligonucleotides, or peptides. With the advancement in biology, designing molecules to inhibit these receptors has become a lot easier and relatively smaller oligonucleotides or peptides have also been explored. Currently in the market, many drugs targeting the receptors are biologics such as mAbs and recombinant/fusion protein [34,35,36]. Viz, TNF blockers (Adalimumab, Certolizumab pegol, Etanercept and Infliximab), IL1R antagonist (Anakinra, Canakinumab), CD30 chimeric mAbs (Brentuximab vedotin), RANK ligand inhibitors (Denosumab). TNFR and IL1R mAbs are well-established and approved for various clinical indications, whereas in small molecule inhibitors, there is only one preclinical candidate. UCB-6786 by UCB pharma, a TNFR small molecule inhibitor, has only been tested for in vivo efficacy in collagen antibody induced arthritis mice model [37]. 

TLR signaling has been immensely studied for several years due to its indispensable role in various disease conditions such as cancer, inflammatory and autoimmune diseases. Both TLR agonist and TLR antagonist biologics have been testing extensively in clinical trials [38]. Focusing small molecule inhibitors, some TLR 4 and TLR7/8/9 antagonists have successfully entered various stages of clinical trials (Table 1). TLR7/8/9 antagonists are mostly antisense oligonucleotide (ASO) like Bazlitoran (IMO-8400), which was granted orphan drug status [39] for diffuse large B cell lymphoma (DLBCL) and Waldenstrom’s Macroglobulinemia (WM). However, further studies were suspended for both indications [40]. Other ASO molecules such as IMO-3100 and IMO-9200, also did not go beyond phase 2 and phase 1 stage respectively. Only one small molecule TLR7/8/9 antagonist, CPG-52364 developed by Coley Pharmaceutical Group was taken into phase 1 trial for systemic lupus erythematosus (SLE) but was discontinued in 2010 (https://clinicaltrials.gov/ct2/show/NCT00547014). TLR4 antagonist, JKB-121, a small molecule inhibitor developed by TaiwanJ Pharmaceuticals for the non-alcoholic steatohepatitis (NASH) indication, is in jeopardy after it failed to demonstrate efficacy in a phase 2 placebo-controlled trial due to surprising positive response in the placebo arm [41]. In January 2019, JKB-122, another small molecule inhibitor of TLR 4, was reported to produce positive results from a phase 2 clinical trial evaluating its efficacy and safety during the treatment of patients with refractory autoimmune hepatitis. Moreover, the drug was demonstrated to be safe and well-tolerated in this study. As an orphan drug designation, the study results of JKB-122 provide a further opportunity for the autoimmune hepatitis (AIH) treatment [42] (Table 1).

Many TNFRSF inhibitors are successfully undergoing clinical trials for various indications including RANK ligand (RANKL) inhibitors. RANK signaling essential for the activation of osteoclasts, is mediated through the activation of the c-jun N-terminal kinase (JNK) and NF-κB pathways. Although RANK is activated by RANKL [43], it has been reported that even overexpression of RANK may itself activate the NF-κB pathway. Modulating the RANKL-RANK signaling axis has been a key strategy for diseases like osteoclastogenesis, bone destruction and even for many cancers [35]. Denosumab is a human recombinant monoclonal antibody against RANKL that has achieved orphan drug status [44] for bone cancer and malignant hypercalcemia and is currently marketed for these disease conditions along with other bone disorders, bone metastases, corticosteroid-induced osteoporosis, and male osteoporosis [45,46]. It is currently in phase 3 for breast cancer and non-small cell lung cancer (NSCLC) but there are concerns of increased risk of spontaneous multiple vertebral fractures upon denosumab discontinuation that has to be managed by bisphosphonate [47]. Most other molecules in clinical trials are also antibodies or nanobodies like JMT-103, IBI-307, and ALX-0141. Osteoprotegerin (OPG), a natural peptide that binds to the cytokine RANKL and prevents it from binding to RANK, has also been used to modulate this pathway. Amgen explored recombinant osteoprotegerin (AMGN-0007) but discontinued further studies on this molecule after incidents for bone disorders, cancer pain, postmenopausal osteoporosis, and RA. Similarly, Teva was developing an OPG analogue, CEP-37251, discovered by EvoGenix, and took it to phase 1 for osteoporosis but it was terminated. There is no small molecule inhibitor of RANKL in clinic, but with a better understanding of PPI disrupters, we expect to see one soon [48]. Similarly, other TNFRSF such as BAFF, LTβ, CD27, CD30, CD40, OX40, and Fn14 also do not have any small molecule inhibitor in development.

**Table 1:** Clinical small molecule inhibitors of cell membrane receptors in NF-kB pathway.

Drug	Originator/Developer	Stage	Indication	Trail No.	Other Information
***Toll like Receptor 4 (TLR4) antagonist***					
Ibudilast (MN-166)	MediciNova Inc	Marketed in Japan	Allergic conjunctivitis, Asthma, Cerebrovascular disorders	NCT01860807; NCT04057898	Nonselective phosphodiesterase inhibitor. Neuroprotective benefit observed in clinic. Common side effects are nasopharyngitis and headache. Nausea and depression at higher doses.
		Phase3	Amyotrophic Lateral Sclerosis	NCT04057898	
		Phase 1/2	Glioblastoma	NCT03782415	
	MediciNova Inc/NIH	Phase 2	MS	NCT01982942	
JKB-122	Jenken Biosciences/TaiwanJ Pharmaceuticals	Phase 3	Autoimmune Hepatitis	NCT04371718	Out licensed to Newsoara Biopharma for clinical development of JKB-122 and for preclinical research of JKB-133. Successfully achieved primary endpoint in phase 2 Autoimmune Hepatitis study. Study in HCV patients terminated after completing phase 1.
		Phase 2	Autoimmune Hepatitis	NCT02556372	
		Phase 2	Chronic hepatitis C	NCT02293941	
		Phase 2	NASH	NCT04255069	
VB-201 (TLR2/4 antagonist)	VBL Therapeutics	Phase 2	Psoriasis	NCT01001468; NCT01837420	Did not meet primary endpoint in Psoriasis and Ulcerative Colitis [49]. Currently in preclinical trials for Non-alcoholic fatty liver disease.
		Phase 2	Ulcerative colitis	NCT01839214	
JKB-121	Manal Abdelmalek, Duke University/TaiwanJ Pharmaceuticals	Phase 2	NASH	NCT02442687	Failed to demonstrate efficacy due to surprising positive response in the placebo arm and discontinued for NASH.
***Toll like Receptor 7 (TLR7) antagonist***					
CPG-52364	Pfizer	Phase 1	Healthy Volunteers (For SLE)	NCT00547014	Discontinued for SLE in Jan2020.

Abbreviations: ALT—alanine aminotransferase; MS—multiple sclerosis; NASH—Non-alcoholic steatohepatitis; NIH—National Institutes of Health; SLE—systemic lupus erythematosus.

### 2.2. Inhibition of Receptor Adaptor Protein

#### 2.2.1. Bruton’s Tyrosine Kinase (BTK) Inhibitors

BTK and other Tec family of non-receptor tyrosine kinases act as a platform for bringing together a diverse array of signaling proteins. Among the Tec family kinases, BTK is one of the successfully explored targets with almost 50 molecules entering clinical trials and a couple of others launched for multiple indication [50,51]. 

Small molecule inhibitor, Ibrutinib was developed for chronic lymphocytic leukemia (CLL), mantle cell lymphoma (MCL), graft-versus-host disease (GVHD), marginal zone lymphoma (MZL), and WM. However, this molecule was terminated for further evaluation during the studies of many other cancers at different stages, including breast cancer, rheumatoid arthritis (RA), and seasonal allergic rhinitis (SAR). Acalabrutinib is currently marketed for MCL and in collaboration with AstraZenca was recently approved [52] (Nov.2019) for CLL or small lymphocytic lymphoma (SLL). Zanubrutinib was recently granted [53] an accelerated approval by the US-FDA for MCL as a second line of therapy and preregistered for CLL (Table 2).

Many other superior BTK inhibitors are still in different stages of clinical trials (Table 2). Compared to Ibrutinib, Orelabrutinib has a better response rate and superior safety profile in a phase 2 study with relapsed or refractory MCL patients [54]. Tirabrutinib was granted the orphan drug status and is now preregistered for lymphoma [55]. Recently (Dec.2019) safety and efficacy data from a phase 2 study against CLL [56] and long term safety study with this compound in MCL [57] were disclosed. An oral, reversible covalent BTK inhibitor, Rilzabrutinib is currently in phase 3 for pemphigus. Meanwhile, orally active, brain-penetrant SAR-442168 recently [58] met primary end point for phase 2 trials of relapsing multiple sclerosis (MS) and is entering phase 3 trial later this year in relapsing and progressive MS. ABBV-105 is going through phase 2 for RA and SLE, but for better clinical success, it is being explored in combination (renamed as ABBV-559) with JAK1 inhibitor (Upadacitinib) for the same disease (Table 2). 

Dasatinib, a small molecule inhibitor of Bcr-abl tyrosine kinase and Src-Family kinase and later found to be a BTK inhibitor as well, was launched for CLL. It is in different stages for other cancers, but is discontinued for pancreatic cancer [59] and scleroderma due to adverse events and reports of spontaneous pulmonary arterial hypertension [60]. Similarly, EGFR inhibitors (Olafertinib and DZD-9008) were later found to inhibit BTK. BMS-986142 and Fenebrutinib by BMS and Genentech were taken to clinic for many indications, but the studies were terminated or completed without any significant outcome due to various reasons, respectively. Many other small molecule BTK inhibitors like CT-1530, BIIB-068, TAK-020 or GDC-0834 were also discontinued or no development was reported in phase 1 itself (Table 2).

**Table 2:** Clinical small molecule inhibitors of cellular receptors adaptor protein in NF-kB pathway.

Drug	Originator/Developer	Stage	Indication	Trail No.	Other Information
***Bruton’s tyrosine kinase (BTK) inhibitors***					
Zanubrutinib	BeiGene	Launched at 2019	MCL	NCT04002297	BRUKINSA oral dose is 160 mg twice daily or 320 mg once daily. TEAE: Decreased neutrophil count, platelet count and hemoglobin [53]. Case reports of Hepatitis-B reactivation.
		Preregistration	CLL	NCT03734016	
		Phase 3	BCL, RRWM	NCT03332173	
		Phase 2	DLBCL, Follicular lymphoma, Lymphoma, CLL, Lymphosarcoma, FL, MZL	NCT03145064; NCT04282018	
Acalabrutinib	AstraZeneca/Parexel	Phase 3	CLL	NCT04008706	Potentially serious TEAE: Severe bone marrow suppression, infections, bleeding, tumor lysis syndrome, and renal toxicity [61]. Report of Atrial-flutter in an off-label use. Herpes zoster infection and a case of Hepatitis-B reactivation observed.
	Acerta Pharma/AstraZeneca, Biologics Inc, Merck	Launched 2017	MCL	NCT02972840	
	Acerta Pharma	Phase 3	CLL	NCT02970318	
	Acerta Pharma/AstraZeneca	Phase 2	COVID-19	NCT04346199, NCT04380688	
		Phase 2	Chronic GVHD	NCT04198922	
	Acerta Pharma	Phase 2	WM	NCT02180724	
		Phase 2	Metastatic Pancreatic Cancer	NCT02570711	
		Phase 2	RA	NCT02387762	
	Acerta Pharma BV Merck Sharp & Dohme Corp.	Phase 2	Ovarian Cancer	NCT02537444	
		Phase 2	NSCLC	NCT02448303	
		Phase 2	Squamous Cell Carcinoma of the Head and Neck	NCT02454179	
	Acerta Pharma/Swedish Medical centre	Phase 2	DLBCL	NCT03736616	
Ibrutinib	Janssen/Pharmacyclics; M.D. Anderson Cancer Center	Launched 2013	CLL	NCT02801578	420 mg orally once daily dose for CLL. Serious TEAE: hemorrhage, hypertension, ventricular arrhythmias, atrial fibrillation, atrial flutter, decrease in blood cell counts and Tumor lysis syndrome. Invasive fungal infections and Hepatitis-B reactivation observed. Ibrutinib-induced autoimmune hemolytic anemia and rare nail plate abnormalities also seen [62].
		Launched 2013	WM	NCT02165397	
		Launched 2013	MCL	NCT01646021	
		Phase 2	GVHD	NCT02195869	
	Janssen/Pharmacyclics	Phase 3	Metastatic Pancreatic Adenocarcinoma	NCT02436668	
		Phase 3	DLBCL	NCT01855750	
		Phase 3	RRCLL, SLL	NCT01578707	
Dasatinib	BMS/Dana-Farber Cancer Institute	Launched 2006	CML, acute cell lymphoblastic leukemia-lymphoma	NCT00123487, NCT03020030	TEAE: Cytopenias, fluid retention, pleural effusion, dyspnea, gastrointestinal disorders, skin rash, headache and fatigue [63]. Low blood cell count, Pulmonary Arterial Hypertension and Tumor lysis syndrome also seen. Often requires dose interruption and/or dose reduction.
	BMS	Phase 3	Prostatic Neoplasms	NCT00744497	
	BMS/NCI	Phase 2	DLBCL	NCT00608361	
		Phase 2	Rhabdomyosarcoma	NCT0304170	
	BMS/OSI pharma/M.D. Anderson Cancer Center	Phase 2	NSCLC	NCT00826449	
	BMS/Massachusetts General Hospital	Phase 2	CLL	NCT00438854	
		Phase 2	Cholangiocarcinoma	NCT02428855	
	BMS	Phase 2	Breast Cancer	NCT00767520	
	BMS/Jorge J. Castillo, MD	Phase 2	WM	NCT04115059	
Tirabrutinib	Ono Pharmaceutical/Gilead Sciences	Registered	Lymphoma	NCT03162536	TEAE: neutropenia, lymphopenia, leukopenia, and erythema multiforme [64]. Also observed pneumocystis jirovecii pneumonia and interstitial lung disease in a patient at highest dose of 480 mg QD.
	Ono Pharmaceutical/Gilead Sciences	Preregistration	WM	NCT03740529	
Rilzabrutinib	Principia Biopharma	Phase 3	Pemphigus vulgaris	NCT02704429	Orphan drug status for Pemphigus vulgaris and Idiopathic thrombocytopenic purpura.
Evobrutinib	Merck Serono/EMD Serono	Phase 3	MS	NCT04032158	TEAE: Nasopharyngitis and increases in levels of alanine aminotransferase, aspartate aminotransferase and lipase [65].
	Merck Serono/EMD Serono	Phase 2	RA	NCT02784106	
Orelabrutinib	InnoCare Pharma/Beijing	Phase 2	SLE		TEAE: thrombocytopenia and neutropenia [66]. Profile better than Ibrutinib.
			MZL		
ABBV-105	AbbVie	Phase 2	SLE	NCT03978520	Focus is on ABBV-599 which is the ABBV-105/upadacitinib-combination
			RA	NCT03682705	
ABBV-599	AbbVie	Phase 2	RA, SLE	NCT03682705, NCT03978520	
SAR-442168	Principia Biopharma/Sanofi	Phase 2	Relapsing MS	NCT03996291	Common TEAE: Headache, upper respiratory tract infection and nasopharyngitis [67].
Branebrutinib	BMS	Phase 2	RA, SLE, Sjogren’s syndrome	NCT04186871	In phase 1, no serious TEAE observed [68].
TAS-5315	Taiho Pharmaceutical	Phase 2	RA	NCT03605251	Observed decrease in platelet aggregation and prolonged bleeding time in phase 1 [69].
Remibrutinib	Novartis	Phase 2	Asthma, Sjogren’s syndrome, Urticaria	NCT03944707, NCT04035668, NCT04109313	Phase 2 trial in Urticaria suspended due to COVID-19.
BMS-986142	BMS	Phase 2	RA	NCT02638948	TEAE: dizziness and nausea. Increase in Alanine aminotransferase [70].
			Sjogren’s syndrome	NCT02843659	
Fenebrutinib	Genentech	Phase 2	Urticaria	NCT03693625	Higher doses may increase liver enzymes [71].
Poseltinib	Hanmi Pharmaceutical/Eli Lilly	Phase 2	RA	NCT02628028	Phase 2 discontinued as study failed to demonstrate its target effectiveness in the interim results [72].
Spebrutinib	Celegene	Phase 2	RA	NCT01975610	Celgene acquired by Bristol-Myers Squibb.
		Phase 1	CLL	NCT01732861	
DTRMWXHS-12	Zhejiang DTRM Biopharma	Phase 2	DLBCL, R and RCLL, Follicular Lymphoma	NCT04305444	No development reported for B-cell lymphoma.
		Phase 1	MCL	NCT03836768	
		Phase 1	CLL, BCL	NCT02891590	Evaluate the safety, tolerability and PK profile.
CT-1530	Centaurus Biopharma Co., Ltd.	Phase 1/2	B Cell-NHL, CLL, WM	NCT02981745	Discontinued for all indications
REDX08608	Redx Pharma/Loxo oncology	Phase 1/2	CLL/SLL or NHL	NCT03740529	In basal cell cancer, no development reported.
M-7583	EMD Serono	Phase 1/2	MCL, DLBCL, Relapsed/Refractory B Cell Malignancies	NCT02825836	TEAE: Neutropenia, febrile neutropenia and pneumonia [73].
ARQ-531	ArQule/Merck	Phase 1/2	Hematological malignancies	NCT03162536	Well-tolerated through 65 mg QD [74].
Vecabrutinib	Biogen Idec, Sunesis Pharmaceuticals	Phase 1/2	Hematological malignancies	NCT03037645	TEAE: Anemia, headache and night sweats.
TAK-020	Takeda	Phase 1	RA	NCT02413255	TEAE; Abdominal distension, upper abdominal pain, nausea, and headache
BIIB068	Biogen	Phase 1	SLE	NCT02829541	No update beyond phase 1
AC-0058TA	ACEA Biosciences	Phase 1	SLE	NCT03878303	Phase 1 for Autoimmune disorders completed in 2017 and no further progress reported.
SN-1011	Sinomab	Phase 1	Autoimmune disorder	NCT04041544	
BIIB-091	Biogen	Phase 1	Healthy Volunteer (MS)	NCT03943056	Completes phase 1 trial for Multiple sclerosis.
TG-1701	Eternity Bioscience/TG Therapeutics	Phase 1	Healthy Volunteer (NHL, CLL)	NCT04291846	Encouraging safety profile.
CG-806	CrystalGenomics, Aptose Biosciences	Phase 1	CLL, SLL, NHL	NCT03893682	No drug-related dose-limiting toxicities.
***Interleukin-1 receptor-associated kinase (IRAK) inhibitors***					
PF-06650833	Pfizer	Phase 2	RA	NCT02996500	TEAE: Infections and infestations [75].
CA-4948	Curis Pharmaceuticals	Phase 1	AML, MDS	NCT04278768	Adverse events: amylase/lipase increased neutrophil count decreased, rash and rhabdomyolysis [76].
			Hematological malignancies	NCT03328078	
R-835	Rigel Pharmaceuticals	Phase 1	Autoimmune and Inflammatory Diseases		Rigel initiates phase 1 clinical trial [77].
BAY-1834845	Bayer	Phase 1	Pelvic Inflammatory Disease	NCT03054402	No update beyond phase 1 for Pelvic Inflammatory Disease
			Inflammation	NCT03244462	
			Psoriasis	NCT03493269	
BAY-1830839	Bayer	Phase 1	RA	NCT03540615	
			Health volunteers (For RA)	NCT03965728	
***Cellular Inhibitor of Apoptosis Proteins (c-IAP) inhibitors***					
Birinapant (TL32711)	Jonsson Comprehensive Cancer Center, NCI	Phase 2	High Grade Ovarian, Fallopian Tube, Primary Peritoneal Cancer	NCT02756130	Birinapant and pembrolizumab combination had futile outcome in patients with MSS colorectal cancer [78].
	TetraLogic Pharmaceuticals	Phase 1	Hepatitis B	NCT02288208	
APG-1387 (SM-1387)	Ascentage Pharma	Phase 2	Advanced Solid Tumors	NCT04284488	TEAE: Elevated bilirubin, lipase increase and shortness of breath [79]. Exploring for Hepatitis B in both Treatment-experienced and naïve patient.
		Phase 2	Myelofibrosis	NCT04354727	
		Phase 1	Advanced Solid Tumors or Hematologic Malignancies	NCT03386526	
	Ascentage Pharma, HealthQuest Pharma	Phase 1	Chronic Hepatitis B	NCT03585322	
LCL-161	Mayo Clinic	Phase 2	RR Plasma Cell Myeloma	NCT01955434	Hematologic toxicities: thrombocytopenia and anemia [80]. Completed phase I trial in multiple myeloma. No development reported for pancreatic cancer and solid tumors.
	MD Anderson Cancer Center, NCI, Novartis	Phase 2	Myelofibrosis	NCT02098161	
	Novartis Pharmaceuticals	Phase 2	Breast Cancer	NCT01617668	
		Phase 2	Small Cell Lung Cancer Ovarian Cancer	NCT02649673	
	US Oncology Research Novartis, Delta Clinical Research, LLC	Phase 1	Metastatic Pancreatic Cancer	NCT01934634	
	Novartis Pharmaceuticals	Phase 1	Neoplasms	NCT01968915	
		Phase 1	Solid Tumors	NCT01240655	
		Phase 1	Advanced Solid Tumors	NCT01098838	
		Phase 1	MM	NCT03111992	
ASTX660	Astex Pharmaceuticals	Phase 1	AML	NCT04155580	Common TEAE: Anemia, increased lipase and lymphopenia [81].
Debio 1143 (AT-406)	Debiopharm	Phase 1	Advanced Solid Tumors and Lymphomas	NCT01078649	TEAE: Mucositis, dysphagia and anemia [82].
CUDC-427	Curis Pharmaceuticals	Phase 1	Lymphoma	NCT01908413	Few patients discontinued and TEAE includes pruritus and fatigue [83].

Abbreviations: AML—acute myeloid leukemia; BCL—-B-cell leukemia; BMS—Bristol-Myers Squibb; CLL—chronic lymphocytic leukemia; CML—chronic myeloid leukemia; CMML—chronic myelomonocytic leukemia; DLBCL—diffuse large B-cell lymphoma; GVHD—graft versus host disease; HCC—hepatocellular Carcinoma; MCL—Mantle cell lymphoma; MDS—Myelodysplastic Syndrome; MM—Multiple myeloma; MS—multiple sclerosis; MZL—marginal zone lymphoma; NHL—Non-Hodgkin lymphoma; NSCLC—non-small cell lung cancer; RA—rheumatoid arthritis; RRCLL—relapsed and refractory chronic lymphocytic leukemia; RRMM—relapsed and refractory Multiple myeloma; SLL—small lymphocytic lymphoma; SLE—systemic lupus erythematosus; TCL—T-Cell Lymphoma; TEAE—Treatment-emergent adverse effects; WM—Waldenstrom macroglobulinemia.

#### 2.2.2. Interleukin-1 Receptor-associated Kinase (IRAK)

IRAK is the downstream target of TLR and IL1R and the activation of these receptors initiate signaling cascade through MYD88 adaptor protein. This signaling subsequently involving IRAK and IKK complex, leads to NF-κB mediated transcription of certain genes that are involved in various inflammation condition. Quite often, components of this pathway are found to be genetically altered, resulting in specific human cancers [84]. The central role of IRAK in these signaling pathways makes them an attractive target for treating diseases with MYD88 gene mutations as observed in B-cell malignancies [84] and certain inflammatory diseases [85] (Table 2).

The most advanced IRAK small molecule inhibitor, Pacritinib, which primarily targets Janus Kinase 2 (JAK2) and Fms-like tyrosine kinase 3 (FLT3), also inhibits IRAK1 [86]. In 2019, dual IRAK1/4 inhibitor, R-835, successfully completed phase 1 study for autoimmune disorder. Among selective IRAK4 reversible inhibitors, PF-06650833, which is currently at phase 2 for RA and hidradenitis suppurativa, is the front runner and has favorable safety and pharmacokinetic profile from a phase 1 study [75]. Orally active CA-4948 (AU-4948) that was out-licensed to Curis by Aurigene, is currently in phase 1 for acute myeloid leukemia (AML), Myelodysplastic syndromes and non-Hodgkin lymphoma (NHL) [87]. Though, it also inhibits FLT3, it is being showcased as an IRAK4 inhibitor. BAY-1834845 was considered for pelvic inflammatory disorders, psoriasis and RA, but there is no update since the phase 1 trial, which was completed in 2018. BAY-1830839 for RA completed its phase 1 study with increasing single oral doses mid of last year. There were investigations also conducted for safety, tolerability, and pharmacokinetics during increasing repeated oral doses and potential drug-drug interactions with midazolam and methotrexate (Table 2). 

#### 2.2.3. Phosphatidyl Inositol-3 Kinases (PI3K)/AKT 

PI3K, a lipid kinase, mediates signaling from growth factors, cytokines, TLRs, TCR, BCR, and cell stress. The PI3K mediated signaling activates one of its signaling cascades involving the NF-κB pathway through direct activation of IKK via phosphorylation of IKKα [88] and also through AKT (also known as protein kinase B) phosphorylation eventually leading to NF-κB signaling [89,90]. PI3K comprises of one catalytic p110 subunit and two regulatory p85 and p55 subunits. Based on different structures and specific substrates of PI3K, it is divided into three classes called Class I, II, and III PI3Ks and of these Class I are majorly explored. Class I PI3Ks classified as class IA and class IB further Class1A consists three isoforms PI3Kα, PI3Kβ, and PI3Kδ and their catalytic subunits are p110α, p110β, and p110δ, respectively, while p110γ catalytic subunit represents class IB. PI3K P110α is strongly correlated with angiogenesis, while p110β, δ, and γ contribute to inflammatory responses [91,92,93]. 

At basal conditions, the p110 catalytic subunit gets stabilization by forming a heterodimer with regulatory p85 subunit [94,95]. Upon stimulation, phosphorylation p85 regulatory subunit of PI3K leads to the activation of the p110 catalytic subunit. The activated PI3K heterodimer (viz. p110α and p85) induce the conversion of phosphatidylinositol 4,5 bisphosphate (PIP2) to phosphatidylinositol (3,4,5)-trisphosphate (PIP3). PIP3, a second messenger binds to downstream targets and leads into its signaling cascades involving NF-κB pathways through direct activation of IKK via phosphorylation of IKK or through AKT phosphorylation and subsequent activation of p65 through IKK complex [89,90,95,96]. 

Inhibition of PI3K or any downstream target like AKT will effectively modulate the signaling cascade including activation and NF-κB-dependent gene expression. Many PI3K and AKT inhibitors have been taken into clinical trials for a multitude of diseases and are extensively reviewed in the literature [95,97,98]. Some of the PI3K inhibitors are even approved for different indications. Many of them are designed to be active against the specific isoform like Idelalisib and Alpelisib, predominantly active against PI3Kα isoform and thus may have reduced ADR. On the other hand, for better efficacy profile, Copanlisib blocks both PI3Kα and PI3Kδ isoforms and Duvelisib specifically inhibits the Class1A PI3Kδ and Class1B PI3K. To date, no AKT inhibitor has been approved in the market but many are in clinical trials [92,95].

#### 2.2.4. Cellular Inhibitor of Apoptosis Proteins (c-IAP)

IAPs comprise of a family of eight member proteins that are well known for their ability to prevent apoptosis through inhibiting caspase activation or activity. Their role as anti-apoptotic proteins and their dysregulation, either by overexpression or loss of endogenous antagonists, is associated with tumor growth, poor prognosis, and resistance to treatment, making them attractive targets [99]. Recent discoveries have shown that cIAP1, cIAP2, and X-linked IAP (XIAP) also regulate signaling pathways of the innate immune system by ubiquitylating their substrates. Moreover, cIAP1 and cIAP2 inhibit the TNF receptor mediated apoptosis through blocking the caspase-8 activation by TRAF2 interaction [100]. 

In TNF-α induced inflammatory pathway, cIAP1/2 plays a vital role in NF-κB activation. Binding of TNF-α to TNFR recruits TNFR-associated death domain protein (TRADD), receptor-interacting protein kinase 1 (RIPK1), TRAF2, and cIAP1/2 to form complex for NF-κB signaling [101]. IAPs have a carboxy-terminal RING (really interesting new gene) domain which allows them to act as ubiquitin E3 ligases that facilitate to conjugate themselves and with associated proteins such as RIPK1 through K11, K48, and K63-linked ubiquitin chains. This ubiquitin chain facilitates the docking of TAB2/3/TAK1 and the IKK subunit NF-κB essential modulator (NEMO), eventually leading to NF-κB dependent and mitogen-activated protein kinase (MAPK) dependent inflammation, proliferation, and cell survival [102]. Apart from inhibiting caspase activation, ubiquitin E3 ligases property of cIAP1/2 encouraged the researcher to explore cIAP1/2 in an immune and inflammatory target [100,101,103,104]. 

ASTX660, a non-peptidomimetic small molecule antagonist of cIAP1/2 and XIAP (X-linked inhibitor of apoptosis protein), is in phase 1/2 study for advanced solid tumors and lymphomas and phase 2 study for Peripheral and Cutaneous T-Cell Lymphoma (TCL) [105] (Table 2). Second mitochondria-derived activator of caspase (SMAC) mimetic, AT406 (Debio-1143), the small molecule IAP antagonist, based on clinically compelling phase 2 results (by Debiopharm) recently (February 2020) got breakthrough therapy status in head and neck cancer [82]. AT406 also achieved orphan drug status for Ovarian cancer [106] and is in phase 2 for solid tumor. Another SMAC mimetic, APG-1387, inhibits cIAP1/2 and XIAP and got approved in February 2020 for phase 1b/2 trial in China for solid tumors [107]. LCL-161 didn’t progress beyond phase 2 trials for breast cancer, multiple myeloma (MM) and myelofibrosis but a recently concluded phase 1 combination study for MM (https://clinicaltrials.gov/ct2/show/NCT03111992) suggest that Novartis is still interested in this molecule. Meanwhile, combination studies of Birinapant (SMAC mimetic) with Keytruda for MSS- CRC (Phase 2) and Entevir/Tenofovir for viral hepatitis B (Phase 1) were terminated due to futility analysis of data [78] and cranial nerve palsies [108], respectively (Table 2).

## 3. Inhibition on IKK Complex in NF-κB Pathway

### 3.1. IKKα and IKKβ Inhibitors

During the stimulation of the canonical NF-κB pathway, IKK complex, made of IKKα and IKKβ as kinase subunit and IKKγ as regulatory subunit, is activated [109]. Activation of the IKK complex plays a central role in the canonical NF-κB pathway. The signaling cascade begins through the activation of receptors such as IL1R, TNFR, TLR, TCR and BCR at the cell membrane level. Once activated, these receptors and their associated protein complexes come together and percolate into intracellular signaling networks by utilizing adaptor protein interactions, protein phosphorylation, non-degradative ubiquitination and other signal-transducing mechanisms to activate this pathway. Evidence shows that diverse signaling cascades from various cellular receptors converge to activate TAK1, which then phosphorylates IKKβ at T-loop serine residues, S177 and S181, resulting in the activation of the IKK complex. IKK complex eventually phosphorylates IκB leading to p65:p50 release [33,110,111]. For the non-canonical pathway IKKα plays an indispensable role. Once TNFRSF receptor (RANK, BAFFR (B-cell activating factor receptor), CD40, TWEAK (Tumor necrosis factor-like weak inducer of apoptosis), and LTβ) is activated in the non-canonical NF-κB pathway, NIK levels are stabilized. NIK phosphorylates IKKα homodimer on T-loop serine residues, S176 and S180. This activates IKKα complex and leads to P100 processing to p52 through partial ubiquitination [14,112]. Thus, IKKα and IKKβ play a critical role in the NF-κB pathway and their inhibitors would effectively block the whole pathway. It is for the same reason that no IKKα or IKKβ inhibitor has cleared phase 2 studies [21] (Table 3). SAR-113945, an IKKα/β small molecule inhibitor developed by Sanofi and MLN-0415 by Millennium Pharmaceuticals did not meet primary endpoints of phase 2 and safety profile of phase 1, respectively. Also, Merck’s AS-602868 and Leo Pharma’s CHS-828 were also discontinued due to unknown reason (Table 3). Prescott et. al. [109] has discussed these failures and reasoned that the development of more selective non-ATP competitive inhibitors, usage of isoform-specific readouts to differentiate role of IKKα vs. IKKβ in cellular activity and appropriate application of therapeutics should lead to more clinical success.

Sulfasalazine, a disease-modifying anti-rheumatic drug (DMARD) launched for RA and other autoimmune disorders, was also reported to directly inhibit IKKα and IKKβ by antagonizing ATP binding [113]. Anti-inflammatory and immunosuppressive effects of Sulfasalazine are attributed to the suppression of NF-κB activation via inhibition of IKKs. Many nonsteroidal anti-inflammatory drugs (NSAID) like Aspirin and Salicylate are inhibitors of NF-κB pathway [114]. They inhibit ATP binding to IKKβ and thus prevent activation & translocation of NF-κB to the nucleus. Salsalate, the pro-drug of salicylate, is marketed for inflammatory and non-inflammatory disorders. Moreover, in diabetes, since the NF-κB pathway serves as a potential target, clinical trials to test for the efficacy of salsalate in reducing glycemia and insulin resistance diabetes were undertaken successfully [115].

### 3.2. Inhibitor of Nuclear Factor Kappa-B Kinase Subunit Gamma (IKKγ) Inhibitors

IKKγ, also known as NF-κB essential modulator (NEMO), is the regulatory subunit of the IKK complex. Its inhibition or inhibition of its binding to the complex renders IKK inactive. Unlike inhibition of IKKα or IKKβ that results in gross toxicity, inhibition of IKKγ only affects the NF-κB pathway mediated by IKK complex and will not inhibit the standalone activities of IKKα or IKKβ or the non-canonical pathway. NEMO Binding Domain (NBD) peptide was developed to inhibit binding of NEMO to IKKα or IKKβ and reportedly inhibits only the inflammation induced NF-κB activation pathway, with little or no effect on the basal NF-κB activity [21,116]. Unfortunately, due to the low plasma stability and druggability issues of this peptide, it was never taken to clinic. Even modifications of NBD [117] such as covalently linking it to either cell penetrating peptides or Drosophila Antennapedia domain or even macrocyclization to make it into a proteolytically stable bicyclic peptide could not take any NEMO inhibitor to clinical trials. To succeed, IKKγ inhibitors have to overcome the proteasomal degradation and address drug delivery issues.

### 3.3. IKKε and Tank Binding Kinase 1 (TBK1) Inhibitors

IKKε share 67% sequence identity with TBK1 and collectively regulates activation of interferon regulatory factor (IRF)-family factors (IRF3/5/7) by Rig-I like receptors and other receptors [118]. Phosphorylation of IRF3 has subsequently led to expression of interferon-stimulated genes (ISGs). TBK1 also phosphorylates STING (stimulator of interferon genes) and makes it more accessible for binding to IRF3. To date, no molecule that was specifically designed to inhibit IKKε or TBK1 has reached clinic trials. However, a number of small molecules that have been approved or explored for different mechanism of actions, were later found to inhibit IKKε/TBK1 [119]. Amlexanox (Histamine and Leukotriene inhibitors—approved for Asthma), BX-795 (three phosphoinositide dependent protein kinase 1 inhibitors), Momelotinib (JAK inhibitor) are some of the examples. These compounds led to specific inhibitors like MPI-0485520 (developed by Myrexis) and DMXD-011 (by Domainex) but they were not taken beyond preclinical studies. Preclinical research for their use as a synthetic lethal target and their combination therapy are being explored and we may see more molecules in clinical trial.

**Table 3:** Clinical small molecules inhibitors targeting IKK complex in NF-κB pathway.

Drug	Originator/ Developer	Stage	Indication	Trail No.	Other Information
***IKK α/β Inhibitors***					
CHS-828	Leo Pharma	Discontinued at phase 2	Solid tumor	NCT00003979	Originally Nicotinamide phosphoribosyl transferase (NAMPT) inhibitor. Dose-limiting toxicities; thrombosis, thrombocytopenia, esophagitis, diarrhea, and constipation [120].
IMD-1041 (Pro-drug of IMD-0354)	Institute of Medicinal Molecular Design	Phase 2	COPD	NCT00883584	No further development reported for COPD. Clinical phases for other indication unknown.
		NA	Age-related macular degeneration, DM, Glaucoma, PF	NA	
SAR-113945	Sanofi	Discontinued at Phase 2	OA	NCT01598415	SAR-113945 gave as intra-articular injection. Phase 1 studies was promising but Phase 2b proof-of-concept study failed to show efficacy in a larger patient sample size [121].
		Discontinued at Phase 1	OA	NCT01113333, NCT01463488, NCT01511549	
MLN-0415	Millennium Pharmaceuticals	Discontinued at Phase 1	Arthritis, Inflammation, MS	NA	Unfavorable safety profile in Phase 1.
VGX-1027	VGX Pharmaceuticals	Phase 1	Healthy subjects (RA)	NCT00627120	No development reported for RA or other diseases.
		Phase 1	Healthy subjects (RA)	NCT00760396	
Teglarinad Chloride (EB-1627; GMX1777)	Leo Pharma	Phase 1	Malignant melanoma	NCT00724841,	Multi dose study conducted with combination of Temozolomide.
		Phase 1	Lymphoma, Solid tumors	NCT00457574	Single therapy was performed.
AS-602868	Merck	Discontinued at Phase 1	Hematological malignancies	NA	Also, inhibit FLT3.

Abbreviations: COPD—chronic obstructive pulmonary disease; DM—Diabetes mellitus; FLT3—FMS-like tyrosine kinase 3; MS—Multiple sclerosis; NA—not available; OA—osteoarthritis; PF—Pulmonary fibrosis; RA—rheumatoid arthritis.

### 3.4. NF-κB Inducing Kinase (NIK) Inhibitors

NIK is the master regulator of non-canonical NF-κB pathway [18]. It phosphorylates IKKα and that subsequently initiates p100–p52 processing. Mature p52 heterodimerizes with RelB and translocate to the nucleus to regulate gene transcription. Stabilization of NIK and its accumulation is a hallmark in many cancers. Further, the relevance of the non-canonical NFKB pathway in B-cell maturation makes NIK a very desirable target. Many research groups, including pharmaceutical companies like Amgen, Genentech published multiple patents with subnanomolar enzymatic activity and even appreciable in vivo efficacy. However, none of them made it to clinical trials. The closest one was Tracon pharmaceutical’s TRC-694 (JNJ-64290694 in-licensed from Janssen) that they were planning to take into phase 1 proof-of-concept study for patients with hematologic malignancies, including myeloma. Last year they returned the rights to Janssen after completion of the pre-clinical study and no further development were reported [122]. Most scaffolds, for potent NIK inhibition have an alkyne side chain that may be associated with some toxicity, but structurally different compounds published [123] by Johnson not being considered for clinical trial negate this theory. The probable biology of NIK and its exact role in disease are still grey areas and appropriate indication or relevant combination needs to be worked out for clinical consideration.

## 4. Molecules that Inhibit Ubiquitin-Proteasome System (UPS)

The relationship between protein homeostasis and proteolysis plays a key role for regulation of many pathways including NF-κB pathway. UPS is one of the main mechanisms [124] of intracellular protein degradation that is also important for activating protein by partial degradation or post-translational modification. Proteolysis is highly controlled [125] by multiple steps, both at the proteasome end and the targeted protein for degradation, to avoid non-specific degradation. It involves diverse enzymes that render further specificity to the proteolytic degradation. Some proteins need not be ubiquitinated before degradation like conversion of p105 to p50 in canonical NF-κB pathway or in post-translational processing. Similarly, some proteins also undergo nondestructive polyubiquitination in the signaling cascade.

In UPS, covalent attachment of multiple ubiquitin molecules with substrate proteins are intended for proteasomal degradation, which gives a recognition signal for the 26S proteasome. The two distinct and successive steps are involved in the degradation of protein substrates in this pathway, the first one is the ubiquitin conjugation cascade and the second one is destruction process mediated by proteasome core. The former one encompasses the enzymes required for activation, conjugation, and ligation of ubiquitin to protein substrates and the later one takes ubiquitinated proteins to their final fate. Ubiquitination happens typically with three sets of enzymes such as ubiquitin activating enzymes (E1), ubiquitin conjugating enzymes (E2), and ubiquitin ligases (E3) [126,127].

In the first step, E1 activates this cascade through adenylation of the ubiquitin at the terminal carboxyl group of Glycine involves the hydrolysis of ATP to PPi. Eventually, E1 covalently links to ubiquitin via a high-energy thioester linkage. As a second activity, the activated ubiquitin is transferred from E1 to conjugating enzyme E2. On the final step, typically ubiquitin to the protein substrate requires E3 or ligase via forming an amide isopeptide bond between the carboxyl group of Glycine of ubiquitin and amino group of the protein substrate’s internal Lys residue. After the mono-ubiquitination, subsequent ubiquitination conjugation cascade happens between glycine residue of ubiquitin and lysine residue of ubiquitin that is already conjugated to the protein substrate called as poly-ubiquitination [128].

Protein substrate modification in UPS comprises two types of ubiquitin chains, single linkage types are called homotypic chains (mono and polyubiquitylation), where heterotypic chains comprise mixed linkages within the same polymer or one ubiquitin molecule has two or more ubiquitylated sites (mixed and branched ubiquitylation). Proteins can be modified at one lysine residue with either a single ubiquitin molecule or ubiquitin polymers called mono and polyubiquitylation respectively, multiple lysine residues modified with a single ubiquitin known as multiple mono ubiquitylation [126,129].

Heterotypic chains are classified into mixed chains or branched chains. In mixed chains, each ubiquitin is modified only once by another ubiquitin, wherein branched chains, each ubiquitin can be modified by two or more ubiquitin molecules. Due to the huge number of possible conjugate combinations in branched chains, it is affecting different signaling pathways and K48/K63 is one of the most studied branched chains, implicated in NF-κB signaling and apoptosis [128,129,130].

### 4.1. Proteasome Inhibitors

In the canonical and non-canonical NF-κB pathways, proteolytic degradation modulates multiple steps [125]. In the canonical pathway, proteasomal degradation of IκB leads to release of active NF-κB complex (p50/RelA). In the non-canonical pathway, NIK/IKKα mediated phosphorylation of p100 converts it into p52 through partial proteolysis. Thus, these can be targeted for therapeutic intervention and proteasome inhibitors can be used to effectively modulate the NF-κB pathway (Table 4). On the other hand, the critical role played by the proteasomes in fundamental cellular processes, may also lead to target related ADR.

The first clinically approved small molecule proteasome inhibitor, Bortezomib with boronic acid functionality, is a slow reversible inhibitor of the 20S proteasome [131]. It is currently marketed for MCL, MM, and WM and registered for B-cell lymphoma (BCL). It is undergoing phase 3 for DLBCL and relapsed, or relapsed and refractory multiple myeloma (RRMM) (Table 4) [132]. The poor tissue penetration and high plasma clearance made it a poor choice for treating solid tumors [133]. Development of resistance and peripheral neuropathy are serious limitations of this first-in-class drug [131]. Ixazomib, a boronic ester pro-drug, was the first orally administered drug [134] approved by the FDA in 2015. Though it is also a reversible inhibitor, unlike Bortezomib, it had fast disassociation rate when bound to RBC and had better tissue distribution. It was developed by Takeda and received orphan drug status for MM and amyloidosis (Table 4). Ixazomib was approved in combination with lenalidomide and dexamethasone for RRMM [135]. Last year, phase 3 trial of combination with dexamethasone was discontinued, as it did not demonstrate significant improvement in overall hematologic response compared with standard therapy in patients with relapsed/refractory systemic light-chain amyloidosis [136]. It was also discontinued for solid tumors (Table 4).

The second generation Carfilzomib has an epoxyketone group, making it an irreversible inhibitor of 20S proteasome. It has received orphan drug status for MM and WM. The FDA approved it in 2012 for MM patients who has already received other therapies (Table 4) [137]. Amgen recently also completed phase 2 trial for MM as second-line or greater combination therapy with dexamethasone. Unlike bortezomib, patients using carfilzomib had less severe peripheral neuropathy [137], but showed varying degree of adverse cardiovascular events [138], due to its effects on myocardial proteasomes (Table 4). There were reports [137,139] of severe acute kidney injury and rhabdomyolysis in patients with additional complication. To overcome intravenous administration of Carfilzomib, orally bioavailable Oprozomib was developed. Oprozomib received orphan drug status for MM and WM [140] (Table 4). Last year Amgen and Onyx Pharmaceuticals completed phase 1b/2 trial for MM and hematological malignancies respectively. Earlier, Amgen reported phase 1b/2 combination study with melphalan and prednisone in transplant ineligible patients with newly diagnosed MM [140].

Marizomib is a naturally occurring broad-spectrum proteasome inhibitor isolated from the marine actinomycetes *Salinispora tropica* a cytotoxic constituent called salinosporamide A. It is reported to be less toxic, more efficient and structurally different from other approved proteasome inhibitors (Table 4) [141]. Celgene explored this for the treatment of RRMM and demonstrated clinically relevant activity, good tolerability with no severe peripheral neuropathy or hematologic toxicity in phase 1 trial. Phase 2 trial for RRMM was also encouraging and as it can pass through the blood–brain barrier. This initiated phase 3 for the potential treatment for glioblastoma in combination with temozolomide-based radiochemotherapy [142]. Disulfiram, launched in 1951 to support the treatment of chronic alcoholism, showed anticancer activity, possibly due to the disruption of the NF-κB pathway by proteasomal inhibition [143,144]. It received orphan drug status for glioblastoma and is currently in phase 2 trials for pancreatic cancer as a second-line of therapy (Table 4).

**Table 4:** Clinical small molecules inhibitors targeting Ubiquitin-Proteasome System (UPS) in NF-κB pathway.

Drug	Originator/Developer	Stage	Indication	Trail No.	Purpose/Other Information
***Proteasome Inhibitors***					
Disulfiram	National Institute for Health and Welfare, Finland	Launched 1951	Alcohol dependence	NCT00435435	Acetaldehyde dehydrogenase Inhibitor Investigated disulfiram and copper-supplement as add-on treatment. Got orphan drug status for Glioblastoma. Addition of disulfiram to chemotherapy in NSCLC led to 30% decrease in the sum of the longest diameter of target lesion. Common side effect: Bad taste in mouth, headache, nausea, drowsiness. May show hypophosphatemia cases of hepatitis, optic and peripheral neuritis [145].
	Sahlgrenska University Hospital, Sweden	Phase 3	Recurrent Glioblastoma	NCT02678975	
		Phase 3	Lung Cancer	NCT00312819	
	University of Utah	Phase 2	refractory disseminated malignant melanoma	NCT02101008	
	National Cancer Institute (NCI), Slovakia	Phase 2	HER2 negative breast cancer	NCT04265274	
	NCI, Slovakia	Phase 2	Germ Cell Tumor	NCT03950830	
	Mayo Clinic; NCI	Phase 1	Metastatic Pancreatic Cancer	NCT02671890	
Bortezomib	Millennium/Takeda Pharmaceuticals	Launched 2003	MCL, MM, WM	NCT00722137, NCT00257114, NCT02844322	First clinically approved proteasome inhibitor, called VELCADE. Peripheral neuropathy is the common dose limiting toxicity. Thrombocytopenia observed but able to cure by platelet transfusions [146]. Other common issues are gastrointestinal disturbances and fatigue [132].
	The Rogosin Institute	Phase 4	Chronic Kidney Disease and IgA Nephropathy	NCT01103778	
	Melanoma Institute Australia	Phase 4	Melanoma	NCT02645149	
	Janssen Research & Development, LLC	Phase 3	Amyloidosis	NCT03201965	
	University Hospital Southampton NHS Foundation Trust; Janssen-Cilag Ltd.	Phase 3	DLBCL	NCT01324596	
	Millennium/Takeda Pharmaceuticals	Phase 3	Relapsed or Refractory B-cell NHL	NCT00312845	
	European Organisation for Research and Treatment of Cancer (EORTC)	Phase 3	refractory or recurrent cutaneous TCL	NCT01386398	
	Zhengang Yuan, Eastern Hepatobiliary Surgery Hospital, China	Phase 3	Intrahepatic Cholangiocarcinoma	NCT03345303	
	University Hospital Heidelberg	Phase 2	AML	NCT04173585	
	Millennium Pharmaceuticals	Phase 2	NSCLC	NCT01833143	
	Tianjin Medical University General Hospital	Phase 2	Neuromyelitis Optica Spectrum Disorder	NCT02893111	
	Northwestern University National Heart, Lung, and Blood Institute (NHLBI)	Phase 2	PF	NCT02370693	
	Sidney Kimmel Cancer Center at Thomas Jefferson University	Phase 2	GVHD	NCT00408928	
Ixazomib	Millennium/Takeda Pharmaceuticals	Launched at 2015	MM	NCT03173092	First oral proteasome inhibitor, marketed as Ninlaro. “Breakthrough Therapy” status from the U.S.- FDA for relapsed and/or refractory AL amyloidosis. TEAE: thrombocytopenia, peripheral edema, peripheral neuropathy, nausea, diarrhea, constipation, vomiting, and back pain [135,147].
	Millennium/Takeda Pharmaceuticals	Phase 3	Relapsed or Refractory Systemic Light Chain Amyloidosis	NCT01659658	
	Takeda Pharmaceuticals	Phase 2	Immune Thrombocytopenia and Autoimmune Hemolytic Anemia	NCT03965624	
	Millennium/Takeda Pharmaceuticals	Phase 2	Myeloid and Lymphoid Hematologic Malignancy	NCT03082677	
	Takeda Pharmaceuticals	Phase 2	MCL	NCT03616782	
	Millennium Pharmaceuticals	Phase 2	Kidney Diseases andEnd stage Renal Disease	NCT03213158	
	Millennium Pharmaceuticals/NCI	Phase 2	B-cell NHL	NCT02339922	
Carfilzomib (Kyprolis)	Proteolix/Onyx Pharmaceuticals, AbbVie, Genentech & others, Amgen	Launched at 2012	MM	NCT03934684	Hematologic TEAE: thrombocytopenia and anemia. Nonhematologic TEAE: upper respiratory tract infections, fatigue, nausea, dyspnea, diarrhea, and pyrexia [148,149].
	Amgen, Janssen, LP	Phase 3	RRMM	NCT02412878	
	Onyx Therapeutics, Inc	Phase 2	MCL	NCT02042950	
	SCRI Development Innovations, LLC, Amgen	Phase 2	Neuroendocrine Cancer	NCT02318784	
	Onyx Therapeutics, Inc.	Phase 2	Refractory Renal Cell Carcinoma	NCT01775930	
	Amgen	Phase 2	Metastatic Castration-resistant Prostate Cancer	NCT02047253	
	Fred Hutchinson Cancer Research Center, NCI	Phase 2	Chronic GVHD	NCT02491359	
	M.D. Anderson Cancer Center; Amgen	Phase 1	MCL, TCL, DLBCL	NCT01926665	
Marizomib	Nereus Pharmaceuticals/Celgene	Phase 3	Glioblastoma	NCT03345095	TEAE: fatigue, nausea, diarrhea, vomiting, constipation, dizziness, infusion site pain, back pain, anorexia, anemia and dyspnea. Unlike bortezomib, did not induce the limiting toxicities *viz.* peripheral neuropathy, neutropenia and thrombocytopenia [150].
	Celgene	Phase 2	MM	NCT00461045	
	NCI	Phase 2	Anaplastic Ependymoma	NCT03727841	
	Celgene	Phase 1	NSCLC, Pancreatic Cancer, Melanoma, Lymphoma, MM	NCT00667082	
Oprozomib	Onyx Pharmaceuticals	Phase 1/2	R and/or R MM	NCT01832727	TEAE: Hypotension, thrombocytopenia, anemia and diarrhea [151]. Well tolerated in combinations with pomalidomide and dexamethasone.
	Amgen	Phase 1/2	Advanced HCC	NCT02227914	
	Amgen	Phase 1/2	MM, WM	NCT01416428	
	Amgen	Phase 1	Solid Tumors	NCT01129349	
***Deubiquitination (DUB) inhibitors***					
VLX1570	Vivolux, Mayo Clinic	Phase 1/2	MM	NCT02372240	Death of 2 patients receiving two doses at 1.2 mg/kg due to fatal pulmonary toxicity [152].
***NEDD8 activating enzyme (NAE) inhibitors***					
Pevonedistat	Millennium Pharmaceuticals	Phase 3	AML	NCT03268954	DLT: Transaminase elevation, orthostatic hypotension, rash and elevated alanine transaminase [153]. Cardiac failure and multiorgan failure observed at dose of 147 mg/m^2^. No DLT at doses <50 mg/m^2^.
	NCI	Phase 2	Metastatic Cholangiocarcinoma, HCC	NCT04175912	
	University of Michigan Rogel Cancer Center	Phase 2	NSCLC	NCT03228186	
	NCI	Phase 2	Myeloproliferative Neoplasm	NCT03238248	
	Millennium Pharmaceuticals	Phase 1	Advanced Solid Tumors and Neoplasms	NCT03057366	
TAS-4464	Taiho Oncology	Phase 1/2; Terminated	MM, HNL	NCT02978235	As of Dec 2019, discontinued for most indications.
TAK-243	Takeda Pharmaceuticals	Phase 1	Myelodysplastic Syndrome, AML, CMML	NCT03816319	Phase 1 trial for AML, Myelodysplastic syndrome and CMML initiated in May 2019.
	Millennium Pharmaceuticals	Phase 1; terminated	Advanced Malignant Solid Tumors	NCT02045095	

Abbreviations: AML—acute myeloid leukemia; CMML—chronic myelomonocytic leukemia; DLBCL—diffuse large B-cell lymphoma; DLT—dose limiting toxicity; GVHD—graft versus host disease; HCC—hepatocellular carcinoma; MCL—Mantle cell lymphoma; MM—Multiple myeloma; NHL—Non-Hodgkin lymphoma; NSCLC—non-small cell lung cancer; PF—Pulmonary Fibrosis; RRWM—relapsed and refractory Multiple myeloma; TCL—T-cell lymphoma; TEAE—Treatment-emergent adverse effects; WM—Waldenstrom macroglobulinemia.

### 4.2. Deubiquitination (DUB) Inhibitors

DUB inhibitors are also being explored as therapeutic interventions in specific cancers. DUB is accomplished by proteases that can cleave the isopeptide bond formed during ubiquitination. Vivolux identified a novel Ubiquitine Specific Proteases 14 (USP14) specific inhibitor, VLX1570, and it has entered phase 1/2 trial for MM (Combination therapy, Second-line therapy or greater), but was terminated due to dose-limiting toxicity (https://clinicaltrials.gov/ct2/show/NCT02372240) (Table 4). Multiple groups like Nynex Therapeutics, AbbVie, Ubiquigent have reported promising DUB inhibitors at the preclinical stage, but as of date, none of them have reached clinical trials.

### 4.3. NAE (NEDD8 Activating Enzyme) Inhibitors

NAE is a heterodimeric molecule that catalyzes the formation of neural precursor cell expressed, developmentally down-regulated 8 (NEDD8)—adenosine monophosphate (AMP). NEDD8 is a ubiquitin-like protein that modifies cellular targets in a pathway that is parallel to but distinct from ubiquitin modification [154]. NEDDylation is crucial for the activation of Cullin-RING-E3 ubiquitin ligases that are critical for proteasome-mediated protein degradation. Thus, NAE is a desirable target for intervention as it acts upstream of proteasome and catalyzes the first step in the NEDDylation pathway [155]. Unfortunately, this broad intervention may have associated side effects, and one needs to weigh the benefits to the toxicity ratio before taking them to the clinic.

Pevonedistat, first-in-class NAE inhibitor from Millennium Pharmaceuticals, is an AMP mimetic that forms stable covalent adducts with NEDD8 in NAE catalytic pocket and prevents subsequent NAE reaction. It has received orphan drug status for AML and Myelodysplastic syndromes (MDS). Pevonedistat combination study with Azacitidine was terminated in AML patient, but the preliminary results encouraged Takeda to consider phase 2 trial for high risk MS. They also explored the same combination in phase 3 study for low-risk AML, high-risk Chronic myelomonocytic leukemia (CMML), and high-risk MDS [156] (Table 4). Though, due to serious toxicity at higher doses, pevonedistat dose beyond 100 mg/m^2^ is not being considered for further investigation [153]. Takeda had one more Millennium molecule TAK-243 in phase 1 for relapsed or refractory AML, refractory MDS, and CML (https://clinicaltrials.gov/ct2/show/NCT03816319), but the studies were probably discontinued. Similarly, TAS-4464 by Taiho Pharmaceutical was also discontinued in phase 1 for HM and solid tumors, due to business reasons (https://clinicaltrials.gov/ct2/show/NCT02978235).

## 5. Molecules Inhibiting Nuclear Translocation, DNA Binding and Transcriptional Activation of NF-κB

Nuclear import and cytoplasmic export of NF-κB happens through p65 shuttling between cytoplasm and nucleus. As the shuttling is essential to sustain all p65 mediated transcriptional programs and as it is implicated in various disease conditions such as cancer, inflammatory, and autoimmune diseases, it becomes an attractive therapeutic target. After the release of p65 from IκB by stimulation, arginine and lysine-rich nuclear localization signals (NLS) of p65 interacts first with importin α and later with importin β to form a heterodimer. Ferrying of trimeric cytosolic NLS (cNLS)/importin α/β protein complex into the nucleus is facilitated through importin β interaction with nuclear pore complexes (NPCs). NF-κB activation is one of the highly controlled pathways, following the translocation of p65, the increased transcriptional activity eventually results in a negative feedback oscillatory loop for p65 export from the nucleus [157,158,159].

**Figure 3:**
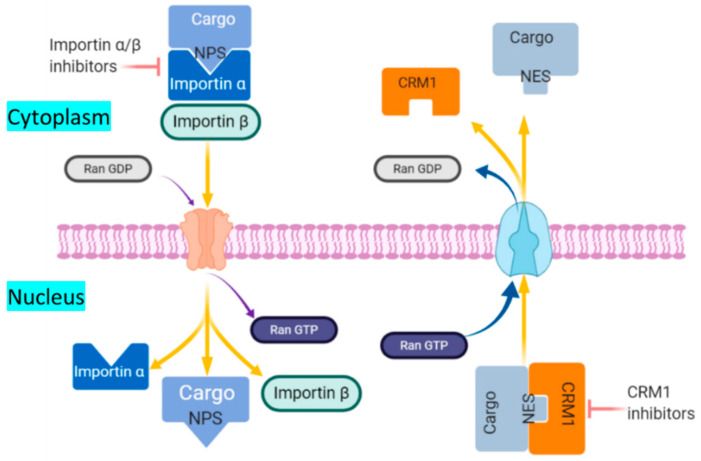
p65 shuttling and the current therapeutic targets explored in the pathway. After ubiquitination IkB, translocation of p65/p50 (Cargo) happens via arginine and lysine-rich NLS (Nuclear localization signals) of p65 interaction with importin α/β heterodimer. This trimeric protein complex (cNLS/importin α/β protein) ferrying into the nucleus is facilitated through importin β interaction with nuclear pore complexes (NPCs). Whereas export of p65 via the CRM1-dependent pathway upon interaction with leucine-rich NES. CRM1 is an export receptor, facilitates the transport of large macromolecules including RNA and protein from the nuclear membrane to the cytoplasm. CRM1 binds the Ran protein bound to GTP, allowing for a conformational change that facilitates cargo protein to nuclear export. Nuclear import and export inhibitors preclude the p65 shuttling via NLS and NES, respectively.

The activated p65 interacts with DNA and stimulates the synthesis of IκBα mRNA and increase the nuclear concentration of IκBα. The increased IκBα protein binds and exports with free p65 and DNA detached p65. This negative feedback regulation is highly controlled by IκBβ and IκBγ. IκBα protein composes a nuclear export regulatory domain contains leucine-rich nuclear export signal (NES) at N terminal region that plays a pivotal role for cytoplasmic localization of p65. The export of p65 happens via the CRM1 (chromosome region maintenance 1/exportin1/Exp1/Xpo1)-dependent pathway upon the interaction of CRMI and IκBα protein at leucine-rich NES domain. CRM1 is an export receptor, facilitates the transport of large macromolecules including RNA and protein from the nuclear membrane to the cytoplasm. CRM1 binds the Ran protein bound to GTP, allowing for a conformational change that facilitates cargo protein to nuclear export (Figure 3) [157,158,159].

The stimuli induced p65 activation is transient, however, it upregulates transactivation of target genes of diverse activities such as cell proliferation and inflammatory cytokine release. Signals between p65 and transcription factors facilitate controlled and efficient transactivation. Further, various post-translational modifications (PTM) including ubiquitination, acetylation, methylation, phosphorylation, and sumoylation of p65 play a vital role in the NF-κB activation outcomes. DNA:p65 binding determines NF-κB activation and it is affected interactions with coactivators and corepressors as well as p65 termination [112,160].

Phosphorylation of p65 has also been implicated in NF-κB regulation and involves mainly serine and threonine sites. The phosphorylation sites are mostly located in the Rel homology domain (RHD) and the transactivation domains (TAD) and the activation results in either increased or decreased transcriptional activity. Histone acetyltransferases (HATs) and histone deacetylases (HDACs) regulate the acetylation of p65 at lysine sites via acetylation and deacetylation respectively. Lysine acetylation of p65 is a reversible process and it depends on coactivators such as p300 and CREB binding protein (CBP) [161]. Tip60 (HIV Tat-interacting protein 60), a coactivator of NF-κB p65, enhances acetylation of Lys310 and upregulate p65 transcriptional activity through PPI. Moreover, Tip60 binds DNA prior to p65 and potentially modulates other cofactors interactions with p65 [162]. The acetylation of specific lysine residue Lys122 [163] and Lys123 decreases DNA binding of NF-κB p65 whereas Lys221 [38] and Lys218 enhances the DNA binding of NF-κB p65. Lys310 acetylation is recognized by the two bromodomains of Brd4, which recruits activated CDK9 to phosphorylate RNA polymerase II for the transcription of a subset of NF-κB target genes [28,164].

### 5.1. Nuclear Export Inhibitors

Leptomycin B (LMB), an irreversible CRM1 inhibitor covalently binds to cysteine 528 and directly blocks its interaction with the NES. LMB was tested for advanced refractory cancer, but was discontinued due to significant systemic toxicity and limited efficiency at phase 1. Later on, Karyopharm developed LMB analogue KOS-2464, but it has not been tested in a clinical setting so far [165,166,167]. Synthetic CRM1 inhibitor (CBS9106) blocks the nuclear export by inducing CRM1 degradation via Neddylation pathway and at present, it is in phase1 trial for metastatic solid tumor. Structure-based drug design led to the further development of CRM1 small molecule inhibitors called as selective inhibitors of nuclear export (SINE) includes Selinexor (KPT-330), Verdinexor (KPT-335) and Eltanexor (KPT-8602) and these are orally bioavailable and highly selective. They are currently in clinical trials for various cancer conditions as standalone or in combination with other drugs. They are also found to be potential antiviral agents against various influenza strains and respiratory syncytial virus. Selinexor (KPT-330) got FDA approval in July 2019 for RRMM and is currently also taken into COVID-19 clinical trial [168,169,170] (Table 5).

**Table 5:** Clinical small molecules inhibitors targeting nuclear translocation, DNA binding, and transcriptional activation of NF-kB.

Drug	Originator/Developer	Stage	Indication	Trail No	Purpose/Other Information
***Selective inhibitors of nuclear export (SINE)***					
Selinexor	Karyopharm Therapeutics	Launched at 2019	RRMM	NCT03110562	Launched for DLBCL in 2020. Side effects include nausea, vomiting, fatigue, diarrhea, decreased appetite, weight loss, thrombocytopenia, neutropenia, and hyponatremia [171]. Thrombocytopenia addressed by platelet transfusions and thrombopoietin receptor agonists. Granulocyte colony stimulating factors were effective at resolving neutropenia. The central nervous system mediated anorexia with weight loss and malaise limits the frequency of dosing to two or three times per week.
		Phase 3	Endometrial Cancer	NCT03555422	
		Phase 2	Thymoma, Advanced thymic epithelial tumor	NCT03193437	
		Phase 2	Coronavirus Infection	NCT04355676	
		Phase 2	Coronavirus Infection	NCT04349098	
		Phase 2	Myelofibrosis	NCT03627403	
		Phase 2	AML (Relapsed/Refractory)	NCT02249091	
		Phase 2	Ovarian, Endometrial and Cervical Carcinoma, Breast Cancer	NCT02025985	
		Preregistered/Phase 2	DLBCL	NCT02227251; NCT03992339	
		Phase 1/2	Diabetic Foot Ulcers	NCT02367690	
		Phase 1/2	NSCLC	NCT03095612	
		Phase 1	Colorectal Neoplasm	NCT02384850	
		Phase 1	BCL	NCT02741388	
		Phase 1	Solid Tumors	NCT02078349	
		Phase 1	Soft Tissue Sarcoma	NCT03042819	
	NCI	Phase 1	Gliosarcoma, Newly Diagnosed Glioblastoma	NCT04216329	
		Phase 1	Recurrent or Refractory Solid Tumors or High-Grade Gliomas	NCT02323880	
Eltanexor	Karyopharm Therapeutics	Phase 2	RRMM, mCRC, mCRPC, HR-MDS	NCT02649790	TEAE: Thrombocytopenia, neutropenia, anemia, leukopenia, and hyponatremia [172].
Verdinexor	Karyopharm Therapeutics	Phase 1	Healthy adults	NCT02431364	Conditional approval by US-FDA to treat canine lymphoma [173].
Felezonexor (SL-801)	Stemline Therapeutics	Phase 1	Solid Tumors	NCT02667873	Felezonexor showed partial response in the interim results for phase 1 study in microsatellite stable colorectal cancer.
***Histone deacetylase inhibitors***					
Vorinostat	Merck	Marketed	Cutaneous TCL	NCT00875056, NCT00091559	TEAE: Thrombocytopenia, dehydration, pulmonary embolism, squamous cell carcinoma, and severe anemia [174]. There are reports of QTc-interval prolongation.
Romidepsin	Celgene	Marketed	Cutaneous and Peripheral TCL	NCT00426764	TEAE; nausea, fatigue, anemia, thrombocytopenia, ECG T-wave changes, neutropenia, and lymphopenia [175].
Belinostat	Onxeo	Marketed	Peripheral TCL	NCT00274651	TEAE: Nausea, fatigue, pyrexia, anemia, and vomiting [176].
Panobinostat	Novartis/Secura Bio	Marketed	MM	NCT01023308	TEAE: Thrombocytopenia, neutropenia, lymphopenia, anemia, diarrhea, fatigue, and nausea [177].
Tucidinostat	HUYA Bioscience	Marketed	Peripheral TCL (different stages for other cancers)	NCT04040491	TEAE: Thrombocytopenia, neutropenia, fatigue, leucopenia, vomiting, diarrhea, nausea, and anemia [178].
***DNA acetylation inhibitors***					
Azacitidine	Pfizer/Celgene	Marketed	AML; CML; MDS	NCT03416179, NCT03416179, NCT01201811	TEAE: Nausea, vomiting, diarrhea, and cytopenia [179]. Myelosuppression is also observed but usually transient.
	AbbVie/	Marketed	MDS	NCT04401748	
Decitabine	Janssen-Cilag/Otsuka Pharmaceutical	Marketed	AML, MDS	NCT02472145, NCT01751867	TEAE: Myelosuppression (neutropenia, thrombocytopenia, and anemia), Febrile neutropenia, pyrexia, fatigue, nausea, cough, petechiae, diarrhea, and constipation [180].

Abbreviations: AML—acute myeloid leukemia; BCL—-B-cell leukemia; CML—chronic myeloid leukemia; DLBCL—diffuse large B-cell lymphoma; HR-MDS—Higher Risk Myelodysplastic Syndrome; mCRC—Metastatic Colorectal Cancer; mCRPC—Metastatic Castration Resistant Prostate Cancer; MDS—Myelodysplastic Syndromes; MM—Multiple myeloma; NSCLC—non-small cell lung cancer; RRMM—relapsed and refractory Multiple myeloma; TCL—T-cell lymphoma; TEAE—Treatment-emergent adverse effects.

### 5.2. Nuclear Import Inhibitors

From the therapeutic view, nuclear export inhibitors are more explored in clinical settings than nuclear import inhibitors and the latter have not entered any clinical trial yet. Ivermectin, a marketed anti-protozoal drug, was later also found to be a specific importin α/β inhibitor. It was reported to show potential antiviral activity against both HIV-1 and dengue virus via inhibiting importin α/β interaction with HIV-1 integrase and NS5 (non-structural protein 5) polymerase proteins respectively [181]. Importazole was reported to be an importin β inhibitor in Xenopus egg extracts and cultured cells in vitro assay [182]. Cell permeable synthetic peptide (SN50) carries a functional domain to inhibit the NLS of p65/p50 and prevent the complex nuclear translocation [183]. Anti-inflammatory peptide-6 (AIP6) showed anti-inflammatory activity in both in-vitro and in-vivo experiments and mechanistically it inhibited p65/p50 translocation and DNA binding [184].

### 5.3. Inhibition of p65 Transactivation and DNA Binding

The p65 has acidic TAD in the carboxyl-terminal end and it is essential for the regulation of the p65 target gene. Activated p65 TAD through phosphorylation enhances protein-protein interaction and interact with 27 various co-transcriptional regulators such as TBP, p300 and ATR etc. [185]. For the therapeutic interventions, competing p65 peptide has been used to encompass the TAD and to inhibit conformational changes that further prevent transactivation of p65. Synthetic peptide, GILZ (glucocorticoid-induced leucine zipper) mimetic and Smad4-binding domain peptide encompasses the TAD and inhibit the transactivation. Currently, there are no small molecule inhibitors available that target p65 TAD. Similarly decoy NF-kB nucleotides bind with p65 RHD which further inhibits the interaction of p65 with cis-elements of the target gene in the DNA. At present, no small molecule is developed to modify interaction [186,187].

### 5.4. Inhibition of Post-Translational Modifications

Various transcriptional factors regulate gene expression through PMTs, where NF-κB is one of the important transcriptional factors regulates gene expression via PMTs. Transcriptional activities of NF-kB are regulated by many PMTs such as acetylation, ubiquitination, phosphorylation, methylation, sulfydration, nitrosylation, and sumoylation. Reversible acetylation is one of the important PMT for p65 and it is mainly regulated by HAT and HDAC enzymes. Acetylation of p65 happens predominately on lysine residue mainly by transcriptional coactivators p300/CBP and some lysine residues by PCAF [163,186,188]. Modulation of reversible acetylation is extensively explored for therapeutic intervention, particularly HAT and HDAC enzymes. To date, many HAT and HDAC inhibitors are approved or in ongoing clinical trials for various indications. Similarly, DNA methylation is another important PMTs regulated by DNA methyltransferases (DNMT) and inhibitors of DNMT are currently in many clinical trials for various indications. For the last two decades, a considerable number of epigenetic-targeted small molecules got FDA approval, such as Azacitidine, Decitabine, Vorinostat, Romidepsin, Belinostat, Panobinostat, and Tucidinostat for various therapeutic indications. Especially HDAC inhibitors studied extensively in drug discovery and few of them approved in the clinic [189] (Table 5).

## 6. Molecules that Can or May be Repurposed as NF-κB Pathway Inhibitors

In addition to the small molecules that were designed to inhibit specific targets in NF-κB pathways, many that were pursued for unrelated targets were later also found to inhibit NF-κB signaling. As discussed earlier, many molecules like Dasatinib, Olafertinib and Pacritinib were originally intended for other targets. It should be noted that in these the intended therapeutic actions may very well be an outcome of synergistic inhibition of all the targets. There are still other classes of compounds where the drugs marketed of an unrelated indication are later found to inhibit NF-κB pathways and may be repurposed for the same. We have already discussed DMARD like Sulfasalazine and NSAID like Aspirin and Salicylate reported to have IKKα inhibitory activity. Gabexate marketed as Anticoagulant was also found to be effective in treating patients with sepsis-associated TNFα mediated disseminated intravascular coagulation. It was proposed [190] to inhibit binding of AP1 and of NF-κB to their respective target inhibiting activation of mitogen-activated protein kinase (MAPK) pathways and prevent proteolytic degradation of IκBα.

Glucocorticoids (Prednisolone and Vamorolone) are well known for their potent anti-inflammatory activities [191] (https://www.reveragen.com/vamorolone/). Their binding to cytoplasmic nuclear hormone receptor (glucocorticoid receptor) was reported to have a transrepression activity and they are also known to repress the action of NF-κB gene activation. Macrolides (Erythromycin and Azithromycin) are also reported [192] for their anti-inflammatory activity as they downregulate cytokine gene expression by inhibiting transcriptional activation of NF-κB. These molecules were originally designed and are being marketed for more than a decade as antibacterial drugs. The activity of these 14-15 member macrolides has propelled research into 12 member analogs that do not show their original antibacterial activity but retain anti-inflammatory properties [193]. Existing know-how of macrolides can assist these analogs in their discovery and development stages.

Polyphenols like curcumin, capsaicin, apigenin, oleandrin, quercetin, resveratrol, cinnamaldehyde, epigallocatechin-3-gallate, etc. are well known for their anti-inflammatory properties [194,195,196] and mostly act through arachidonic acid dependent and independent pathways. While the former involves COX, the latter is generally mediated through NF-κB. They inhibit the NF-κB signaling by either preventing IκB degradation by inhibiting phosphorylation or ubiquitination of relevant kinases or inhibiting the interaction of NF-κB subunits with DNA.

Iguratimod, a disease modified anti-rheumatic drug (DMARD) is known to inhibit the production of immunoglobulins and cytokines and regulate T lymphocyte subsets at the synovial joints [197]. Iguratimod was also reported [198] to inhibit nuclear translocation of NF-κB p65. Mepacrine is an antiprotozoal, antirheumatic, and an intrapleural sclerosing agent. Apart from histamine N-methyltransferase inhibitory activity, it is a DNA intercalating agent that also inhibits the NF-κB pathway [199]. Dimethyl fumerate (one of the active ingredient of Fumaderm) is prescribed for Psoriasis and relapsing multiple sclerosis. One of their mode of action is by interacting with glutathione that leads to the inhibition of NF-κB nuclear translocation and its transcriptional activity [200]. It was reported to inhibit nuclear translocation of NF-κB p50/p65 heterodimers.

## 7. Conclusions and Way Forward

**Figure 4:**
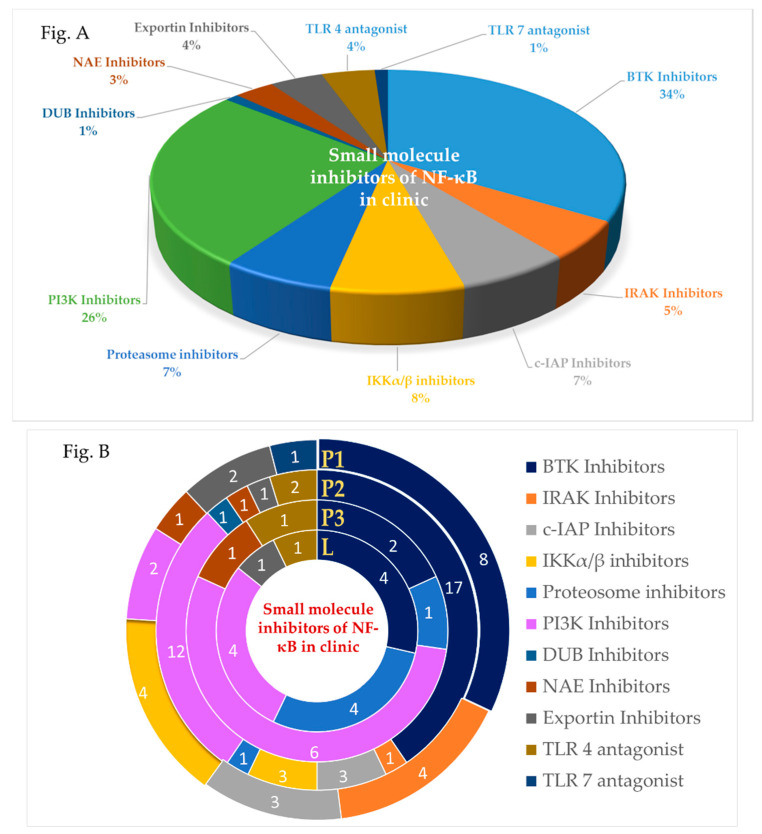
(**A**) Percentage distribution of small molecule inhibitors from various targets in NF-κB pathways reached in the clinic for different indications. (**B**) Similarly, the number of small molecule inhibitors from various targets in NF-κB pathways placed in phase 1(P1), phase 2(P2), phase 3(P3), and launched (L) in clinical trials.

NF-κB pathways play a critical role in almost all chronic diseases and are well-studied and mapped out. They involve multiple players that not only play key roles in signaling, but also are targetable from a drug discovery aspect. This has prompted researchers all over the world to explore thousands of molecules for modulating this pathway. As of date many of these potent drugs are in clinical trials or launched successfully (Figure 4) for diverse therapeutic intervention. Added to this many of the drugs that are currently in use with an unknown or different mechanism was later shown to modulate this pathway also. This is especially true for small molecules that often participate in multiple modes of action. Identification of established drugs showing additional NF-κB activity has led to the repurposing of drugs for new indications and thus further propelled research in this field.

Looking forward, we will definitely see more compounds that inhibit NF-κB pathways that not only will qualify for clinical trials but also get approved for administration in patients. As our understanding of the pathway improves, we will see more focus research in the area of designing inhibitors to their therapeutic area of applications. We anticipate that compounds, instead of getting broad coverage, will be focusing on specific indications or even subtypes. With proper patient stratifications using relevant biomarkers [201], inhibitors of BTK, IRAK, cIAP, PI3K, and even AKT and NAE will see more success in clinical trials. IKK complex inhibitors, one of the main focus of the research due to its central role in NF-κB pathways is challenged by the failure of IKKα/IKKβ inhibitors in the clinic. More selective non-ATP competitive inhibitors, use of isoform-specific readouts to differentiate the role of IKKα vs. IKKβ in cellular activity, and appropriate therapeutic application should lead to more clinical success. Alternatively, inhibition of IKKγ that leads to lower ADR will be more beneficial. The clinical success of IKKγ inhibitors and for other such peptides/pseudopeptides modulated targets, will in turn depend on strategies to overcome the proteasomal degradation and delivery to the site of action. Furthermore, targeting the disease-specific downstream effector of the unregulated NF-κB pathway provides a much safer alternative. Gadd45β (growth arrest and DNA damage 45B)/MKK7 interaction suppresses MKK7/JNK-induced apoptosis and promotes proliferation in cancerous cells. DTP3, a D-tripeptide inhibitor disrupts this interaction and induces apoptosis of cancerous cells [25]. Unlike upstream targets, targeting this should be less toxic to healthy cells. Imperial College London was exploring a Phase 1/2 dose-escalation study in patients with RRMM [202] but not much progress has been reported. For synthetic lethal targets like TBK1 and ATM, clinical trials in disease with specific mutations or studies with relevant combinations are being explored for success. In a few other targets, biology is still in the grey area and their role in modulating the disease, especially in the clinic is yet to be established. NIK is one such example where appropriate indication (beyond MM) or relevant combination needs to be worked out for clinical consideration. Last but definitely not the least, drugs in use that additionally showed NF-κB activity should be actively explored and be used as templates as novel scaffolds.

## Abbreviations

ADR	Adverse drug reaction
AIH	Autoimmune hepatitis
AKT	Protein Kinase B
ALT	Alanine aminotransferase
AML	Acute myeloid leukemia
ATM	Ataxia telangiectasia mutated
BAFF	B-cell activating factor
BAFFR	B-cell activating factor receptor
BCL	B-cell leukemia
BCR	B-cell receptors
BTK	Bruton’s tyrosine kinase
c-IAP	Cellular Inhibitor of Apoptosis Proteins
CLL	Chronic lymphocytic leukemia
CML	Chronic myeloid leukemia
CMML	Chronic myelomonocytic leukemia
COPD	Chronic obstructive pulmonary disease
DLBCL	Diffuse large B-cell lymphoma
DLT	Dose limiting toxicity
DMARD	Disease-modifying anti-rheumatic drug
DUB	Deubiquitination
FLT3	FMS-like tyrosine kinase 3
Fn14	Fibroblast growth factor-inducible 14
GVHD	Graft versus host disease
HCC	Hepatocellular carcinoma
IKK	IκB kinase
IκB	Inhibitor of κB
IKKγ	Inhibitor of nuclear factor kappa-B kinase subunit gamma
IL1R	Interleukin1 receptor
IRAK	Interleukin-1 Receptor-Associated Kinase
IRF	Interferon regulatory factor
JAK	Janus Kinase
JNK	c-jun N-terminal kinase
LT	Lymphotoxin
mAbs	Monoclonal antibodies
MAPK	Mitogen-activated protein kinase
MCL	Mantle cell lymphoma
MM	Multiple myeloma
MS	Multiple sclerosis
MTD	Maximum tolerated dose
MZL	Marginal zone lymphoma
NAE	NEDD8 activating enzyme
NASH	Non-alcoholic steatohepatitis
NBD	NEMO Binding Domain
NEMO	NF-κB essential modulator
NES	Nuclear export signal
NF-κB	Nuclear factor kappa B
NHL	Non-Hodgkin lymphoma
NIH	National Institutes of Health
NIK	NF-κB inducing kinase
NLS	Nuclear localization signals
NPCs	Nuclear pore complexes
NSCLC	Non-small cell lung cancer
OA	Osteoarthritis
ORR	Overall Response Rate
PI3K	Phosphatidyl Inositol-3 kinases
PK	Pharmacokinetic
PDn	Pharmacodynamic
RP2D	Recommended phase 2 dose
RA	Rheumatoid arthritis
RANK	Receptor activator of NF-κB
RANKL	Receptor activator of NF-κB ligand
RRCLL	Relapsed and refractory chronic lymphocytic leukemia
RRWM	Relapsed and refractory Multiple myeloma
RA	Rheumatoid arthritis
RANK	Receptor activator of nuclear factor κ B
SAR	Seasonal allergic rhinitis
siRNA	Small interfering RNA
SLL	Small lymphocytic lymphoma
SLE	Systemic lupus erythematosus
SMAC	Second mitochondria-derived activator of caspase
STING	Stimulator of interferon genes
TAK1	Transforming growth factor beta-activated kinase 1
TBK1	IKKe and Tank binding kinase 1
TCL	T-cell lymphoma
TCR	T-cell receptors
TEAE	Treatment-emergent adverse effects
TLR7	Toll-like receptor 7
TNF-α	Tumor necrosis factor alfa
TNFR	TNF-α receptor
TNFRSF	TNF receptor superfamily
TRAF	TNF receptor-associated factor
TWEAK	Tumor necrosis factor-like weak inducer of apoptosis
UPS	Ubiquitin-proteasome system
WM	Waldenstrom macroglobulinemia.
XIAP	X-linked inhibitor of apoptosis protein
